# The paradoxical role of cytokines and chemokines at the tumor microenvironment: a comprehensive review

**DOI:** 10.1186/s40001-024-01711-z

**Published:** 2024-02-15

**Authors:** Toufik Abdul-Rahman, Shankhaneel Ghosh, Sarah M. Badar, Abubakar Nazir, Gafar Babatunde Bamigbade, Narjiss Aji, Poulami Roy, Hajar kachani, Neil Garg, Lukman Lawal, Zarah Sophia Blake Bliss, Andrew Awuah Wireko, Oday Atallah, Favour Tope Adebusoye, Tetiana Teslyk, Kateryna Sikora, Viktoriia Horbas

**Affiliations:** 1 Medical Institute, Sumy State University, Antonova 10, Sumy, 40007 Ukraine; 2 Institute of Medical Sciences and SUM Hospital, Siksha ‘O’ Anusandhan, Bhubaneswar, India; 3 The University of the West of Scotland, Lanarkshire, UK; 4 King Edward Medical University, Lahore, Pakistan; 5 Department of Food Science and Technology, College of Agriculture and Veterinary Medicine, United Arab Emirates University, Al-Ain, Abu Dhabi, United Arab Emirates; 6 McGill University, Faculty of Medicine and Health Sciences, Montreal, Canada; 7 Department of Medicine, North Bengal Medical College and Hospital, Siliguri, India; 8 Aix-Marseille University, Marseille, France; 9 Rowan-Virtua School of Osteopathic Medicine, One Medical Center Drive Stratford, Camden, NJ 08084 USA; 10 Faculty of Clinical Sciences, University of Ilorin, Ilorin, Nigeria; 11 Centro de Investigación en Ciencias de la Salud (CICSA), FCS, Universidad Anáhuac Campus Norte, Huixquilucan, Mexico; 12 Department of Neurosurgery, Hannover Medical School, Carl-Neuberg-Strasse 1, 30625 Hannover, Germany

**Keywords:** Tumor microenvironment, Cytokines, Chemokines, Tumor, Cancer

## Abstract

Tumor progression and eradication have long piqued the scientific community's interest. Recent discoveries about the role of chemokines and cytokines in these processes have fueled renewed interest in related research. These roles are frequently viewed as contentious due to their ability to both suppress and promote cancer progression. As a result, this review critically appraised existing literature to discuss the unique roles of cytokines and chemokines in the tumor microenvironment, as well as the existing challenges and future opportunities for exploiting these roles to develop novel and targeted treatments. While these modulatory molecules play an important role in tumor suppression via enhanced cancer-cell identification by cytotoxic effector cells and directly recruiting immunological effector cells and stromal cells in the TME, we observed that they also promote tumor proliferation. Many cytokines, including GM-CSF, IL-7, IL-12, IL-15, IL-18, and IL-21, have entered clinical trials for people with advanced cancer, while the FDA has approved interferon-alpha and IL-2. Nonetheless, low efficacy and dose-limiting toxicity limit these agents' full potential. Conversely, Chemokines have tremendous potential for increasing cancer immune-cell penetration of the tumor microenvironment and promoting beneficial immunological interactions. When chemokines are combined with cytokines, they activate lymphocytes, producing IL-2, CD80, and IL-12, all of which have a strong anticancer effect. This phenomenon opens the door to the development of effective anticancer combination therapies, such as therapies that can reverse cancer escape, and chemotaxis of immunosuppressive cells like Tregs, MDSCs, and TAMs.

## Introduction

The past few decades have seen a rapid increase in cancer in many countries globally. With 1,981,030 new cancer cases and 609,360 cancer-related deaths being reported in the United States in 2022 alone [1], it is evident that cancer remains a pressing issue. Extensive research has helped bridge new knowledge concerning cancer mechanisms and devise new therapeutic and diagnostic approaches against different forms of cancers. Nevertheless, much is still unknown about the factors that cause tumors to develop and continuously compromise systems.

**Fig. 1:**
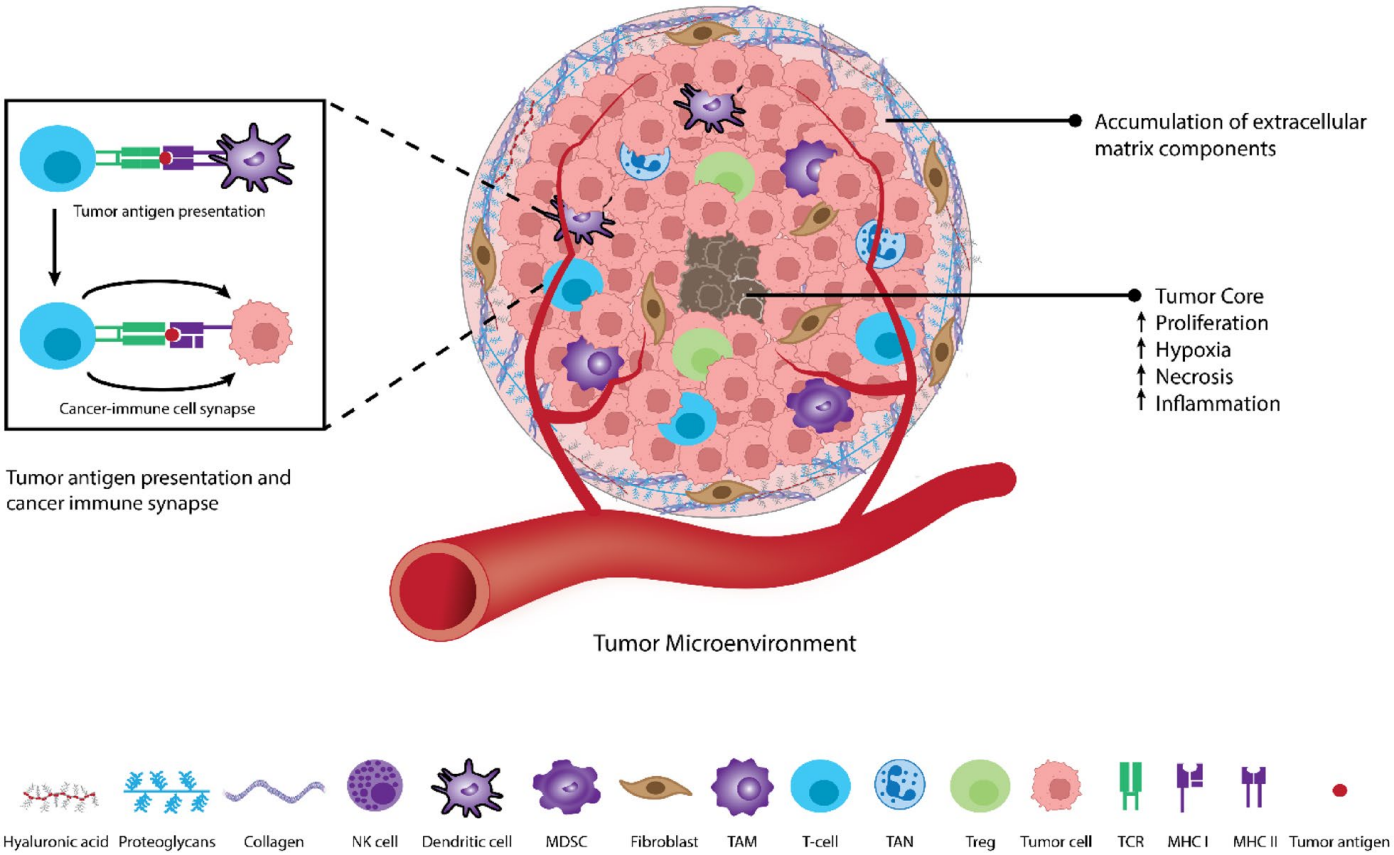
Composition of TME Caption: The TME consists of tumor stromal cells, non-cellular components of the extracellular matrix such as proteoglycans, collagen, hyaluronic acid, etc., and immune cells that surround and interact with tumor cells

The tumor microenvironment (TME) is a complex and ever-changing environment that surrounds and interacts with tumors (Fig. 1). From a cellular perspective, the TME allows cancerous cells to progress and develop through features such as stromal cells, fibroblasts, and endothelial cells alongside adipocytes. These components can then prompt cell survival, local invasion, and metastatic dispersion [2, 3].

Cancer was first described in ancient Egyptian times, but since its discovery, have we truly understood the role different bodily systems play in catering to TMEs and driving cancer progression? The immune system is a potent regulator of the body, which can act as a network portal against pathogens and altered cells [4]. One of the most powerful and provoking immune-related interactions in oncology is the cancer-immune cell synapse, which comprises the connections between cancer antigens and a T cell (Fig. 1) [4, 5]. This synapse is often viewed as controversial due to its ability to promote or suppress cancer progression. However, it is still valuable for its unique role in determining molecular mechanisms that elicit a cancer-immune response [6]. Furthermore, through understanding this synapse, a window of opportunity arises for the development of personalized and improved therapies.

Cytokines are soluble proteins with molecular weights up to 70 kDa, while chemokines constitute a large family of small cytokines, generally with molecular weights ranging from 7 to 15 kDa [7, 8]. Cytokines and chemokines act as central components of both the immune system and cancer-immune synapse, allowing for immune cell trafficking and organization alongside overall immune responses to be regulated and controlled [9]. The dysregulation of these proteins comprises the redundant secreted protein's growth, differentiation, and activation mechanisms. Furthermore, in some instances, these consequences have been reported to exacerbate patients' health by triggering inflammation [10, 11].

The greatest discovery to date concerning these molecules’ contributions to cancer has been the identification of these molecules holding dual relationships to exert their effects rather than acting alone [12]. These prominent twofold abilities can thus further shape mechanisms behind cancer progression or elimination. Therefore, within this review’s scope, the unique roles of cytokines and chemokines at the TME and cancer-immune cell synapse will be explored. In addition, the existing challenges and future opportunities for exploiting these roles to develop novel and targeted treatments will also be addressed.

## Methods

This narrative review on the paradoxical role of cytokines and chemokines at the tumor microenvironment (TME) and cancer-immune cell synapse employed a systematic and comprehensive methodology. A rigorous search of the literature was conducted using well-established databases, including PubMed, Web of Science, Scopus, and Google Scholar. The search string was carefully constructed to incorporate relevant keywords such as "cytokines", "chemokines", "tumor microenvironment", "cancer-immune cell synapse", "tumor progression" and "cancer suppression". This approach ensured the retrieval of pertinent articles. A manual search was also conducted to identify references for recent procedure-specific studies, to enrich the review's content.

Articles were selected based on predefined criteria, including their relevance to the roles of cytokines and chemokines in cancer biology and TME, study types (clinical, preclinical, reviews, and experimental research), publication in English, and a date range primarily focused on the last two decades while also considering earlier seminal works for historical context. Articles that did not align with the review's scope, lacked relevance to cytokines or chemokines, exhibited low scientific rigor, or constituted duplicate publications were excluded. Unpublished studies and stand-alone abstracts were also excluded. This methodological framework lays the groundwork for our narrative review, facilitating a comprehensive examination of cytokines and chemokines' intricate roles in the TME.

## Role of chemokines/cytokines in tumor survival and progression

Tumor progression and eradication has long been a field of interest for the scientific community. Recent discoveries regarding chemokines and cytokines’ role in these processes have led to a renewed urgency in related research. These modulatory molecules play a significant role in tumor development, evasion, proliferation, angiogenesis, metastasis, and replication [13]. Tumor cells can utilize chemokines and cytokines to their advantage allowing for sustained expansion [13]. Malignant growth necessitates the presence of specific environmental and intrinsic factors, such as autonomy of growth, activation of anti-apoptotic pathways, feasible angiogenesis, and invasion, all of which are mediated by cytokines and chemokines and are recognized hallmarks of cancer.

### Immune evasion and recruitment of immunosuppressive cells

**Fig. 2:**
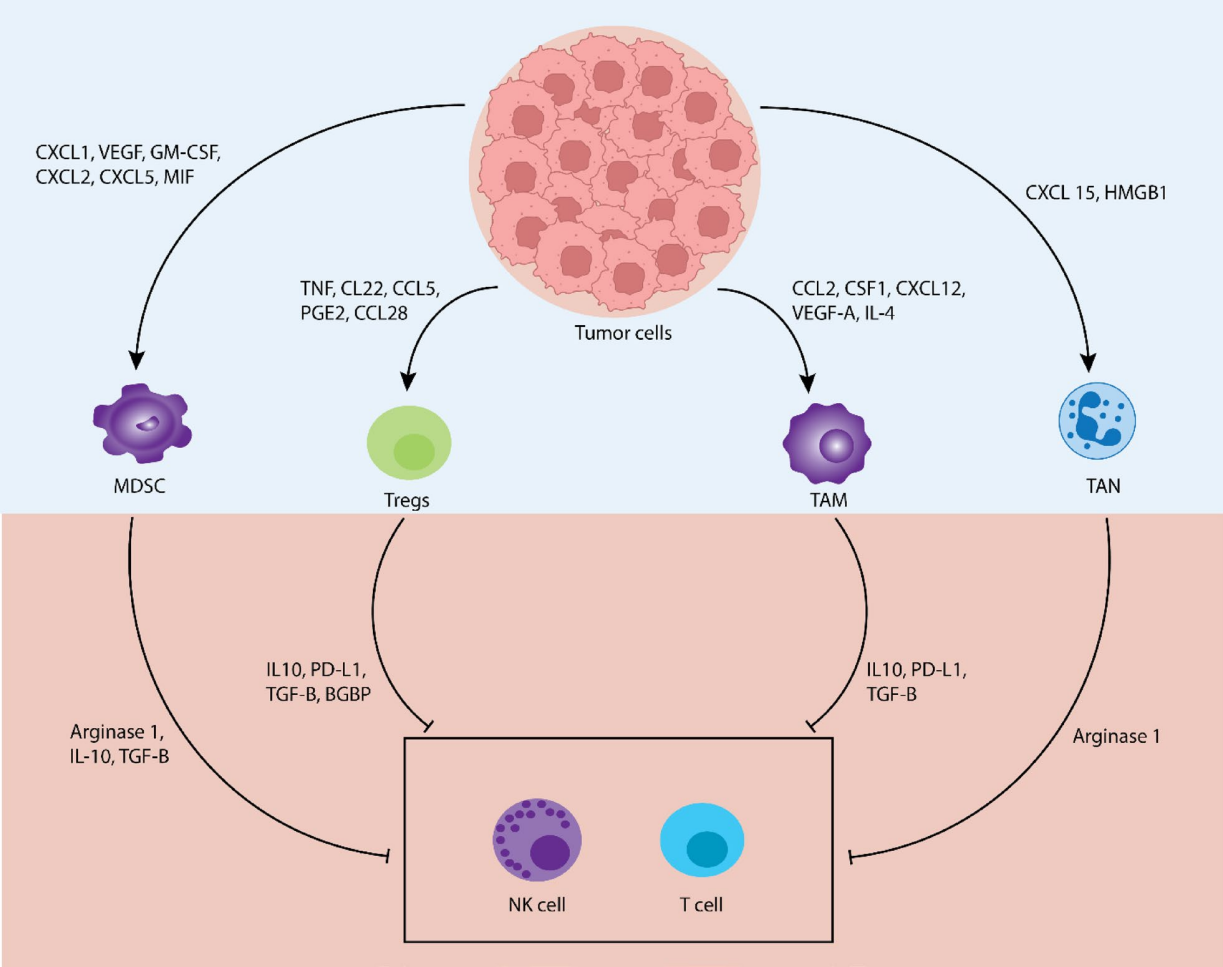
Immunosuppressive roles of cytokines and chemokines in TME. Cytokines and chemokines in the TME play several roles in immunosuppression during tumorigenesis. These include the recruitment of immunosuppressive cells such as MDSCs, Tregs, TAMs, TANs, etc., and inhibition of immune response from T cells and NK cells

Chemokine and cytokine-mediated tumor growth trigger changes in TME, surrounding tissues, and lymphoid organs, leading to altered immune cell activation and further tumor grasp upon the host [14]. One of the ways chemokines and cytokines can mediate this is by facilitating the recruitment of immunosuppressive cells such as myeloid-derived suppressor cells (MDSCs), T regulatory cells (Tregs), tumor-associated macrophages (TAMs), tumor-associated neutrophils (TANs), etc., thus, creating an immunosuppressive TME and inhibiting anti-tumor immune responses carried out by CD4+/CD8+ T lymphocytes and natural killer (NK) cells (Fig. 2).

#### MDSCs

Most TMEs are decorated with immunosuppressive cells such as MDSCs which are capable of inhibiting anti-tumor response and facilitating tumor growth and metastasis. Previous studies have reported the candidature of MDSCs as inhibitors of antitumor immune response and limiting factors in cancer immunotherapy. The presence of MDSCs in tumor-tolerant hosts is characterized by continuous expansion where they contribute to the suppression of antitumor immune response and tumor growth [15]. Different chemotactic factors including Cytokines, chemokines, and complements have all been reported to facilitate the recruitment of these MDSCs in tumor sites. However, chemokines remain the most studied with multiple effects on MDSCs [16–18].

In general, MDSCs are of two types; granulocytic or polymorphonuclear MDSCs (PMN–MDSCs) and monocytic MDSCs (M-MDSCs). They share similarities with neutrophils and monocytes, respectively [15, 19, 20]. The PMN–MDSCs recruitment is carried out by specific chemokines, including CXCR4–CXCL12, CXCR2–CXCL5/CXCL8, while CXCR4–CXCL12, CXCR2–CXCL5/CXCL8, and CCR2–CCL2 chemokines are responsible for M-MDSCs [14]. Among the mentioned chemokines, the expression of CCL2 has been evaluated in different in vivo studies and cancer patients suffering from prostate, breast, ovarian, gastric, melanoma, and renal cell carcinoma (RCC) [15, 21]. The presence of CCL2 in TME was correlated with overall reduced survival of cancer patients and high levels of tumor grades [22]. A comparative study of murine glioma synthesized CCL2 was reported to facilitate the recruitment of CCR2+ Ly6C+ monocytic MDSCs (M-MDSCs) to the tumor site, while CCL2-deficient tumor site witnessed a significant reduction in MDSCs infiltration [22]. Similarly, tumor-associated macrophage CCL2 was reported to facilitate the recruitment of PMN–MDSCs and M-MDSCs in a renal tumor study [23].

Another chemokine with the potential ability to facilitate MDSCs recruitment is CCL12. CCL12 is also known as monocyte chemotactic protein 5 (MCP-5). It is synthesized by different types of cells including immature macrophages and astrocytes, and can attract eosinophils, monocytes, and lymphocytes [15]. High levels of CCL12 have been reported to cause the recruitment of CCR2-induced M-MDSCs to the tumor site of irradiated MC38 colon tumors and the Lewis Lung Carcinoma tumor model [24]. A similar result was observed in the premetastatic lungs of mice with B16F10, HEK-293T, and MS1 tumor cell implants. The presence of CCL12-dependent significantly increases the number of M-MDSCs which enhances tumor cell arrest and metastasis [15].

The generation, migration, proliferation, and maintenance of suppressive properties of MDSCs have also been reported to be induced by tumor-derived and hematopoietic CCR5 ligands like CCL3, CCL4, and CCL5 which can be produced by neutrophils, monocytes, NK cells, T cells, B cells and tumors [25]. High levels of CCR5 ligands correspond to the accumulation of CCR5+ MDSCs in melanoma lesions and tumor progression in human and mouse studies [26]. In addition, elevated levels of CCL5 isolated in breast and cervical cancers have been documented to contribute to tumor stage, relapse, and metastasis [15].

Different suppression mechanisms via effector molecules and signaling pathways for antigen-specific T-cell responses have been documented for PMN–MDSCs and M-MDSCs [15]. Production of large amounts of reactive oxygen species (ROS) including superoxide anion (O2), hydrogen peroxide (H2O2), and peroxynitrite (PNT) is attributed to PMN–MDSCs; however, ROS are highly unstable and short-lived, hence the inhibition of T cells is enhanced by intracellular contact [15]. In addition, PMN–MDSCs suppress immune responses mediated by T cells in antigen-specific mechanisms through increased activity of signal transducer, activation of transcription 3 (STAT 3) and nicotinamide adenine dinucleotide phosphate (NADPH)-signaling pathways [15]. M-MDSCs on the other hand secret nitric oxide (NO), Arginase 1 (Arg1), and immune-suppressive cytokines, such as Interleukin (IL)-10 and transforming growth factor (TGF)-β in abundance. These molecules when compared with ROS stay longer hence, M-MDSCs do not require contact with T cells for tumor progression potentials. The suppression of T and NK cells and recruitment of regulatory T (Treg) cells by M-MDSCs through secretion of CCL3, CCL4, and CCL5 are well-documented in the literature [14]. Unlike PMN–MDSCs, the suppression of T-cell responses by M-MDSCs is established in antigen-specific and non-specific mechanisms through inducible nitric oxide synthase-signaling pathways and STAT 1 with more effective suppression abilities [15, 17, 19].

#### Treg cells

In general, chemokines are released by cancer cells to attract Treg cells within the TME to promote tumor growth. Infiltrated Tregs are clinically significant, because they suppress other immune cells in the TME to support cancer progression [27]. The infiltration of Tregs into the TME is achieved through interaction between chemokines and chemokine receptors. Different chemokines and their ligands such as CXCR3–CXCL9/CXCL10/CXCL11, CCR10–CCL27, CCR6–CCL20, CCR4–CCL17/CCL22, and CCR5–CCL3/CCL4/CCL5 are well-documented in the literature for the recruitment of Treg cells. Treg cells work synergistically with CCR8+ and CCR4+ to facilitate tumor growth by suppressing the responses of T cells in the TME. In addition, Treg cells can facilitate intra-tumoral responses in the presence of the CCR6–CCL20 chemokine axis which consequently promotes Treg cell proliferation [14, 28].

Treg cells exert immunosuppressive effects through several mechanisms. One key mechanism involves the expression of cytotoxic T-lymphocyte antigen (CTLA)-4 on Treg cells. CTLA-4 binds to CD80/86 molecules on antigen-presenting cells (APCs), particularly dendritic cells (DCs), with higher affinity than CD28, thereby inhibiting co-stimulatory signals required for T-cell activation. This interaction can also lead to the physical transfer of CD80/86 from APCs to Treg cells, further hampering T-cell activation through a process called trogocytosis [29]. Inhibitory cytokines such as TGF-β, IL-10, and IL-35, which directly inhibit the activation of effector T cells, are produced by Treg cells [30, 31].

Treg cells can also produce cytotoxic substances like perforin and granzyme, which kill the effector T cells [32]. Treg cells consume IL-2 through their high-affinity IL-2 receptors, limiting the availability of IL-2 for effector T-cell proliferation and activation [33]. Immune checkpoint molecules, including CTLA-4, Inducible T-cell co-stimulator (ICOS), and lymphocyte activation gene-3 (LAG-3), expressed by activated effector Treg cells lead to contribute to the inhibition of cytotoxic functions and proliferation of effector T cells [34]. Enzymes like indoleamine 2,3-dioxygenase (IDO) and tryptophan 2,3-dioxygenase (TDO), which deplete tryptophan in the TME, impair T-cell function. The interaction between CTLA-4 expressed by Treg cells and CD80/86 on APCs can promote the secretion of IDO. Treg cells are also sensitive to oxidative stress due to lower expression of NRF2, a key antioxidant transcription factor, making them prone to apoptosis. Apoptotic Treg cells release ATP, which is metabolized to adenosine by CD39 and CD73, both highly expressed by Treg cells. Adenosine then binds to the A2A receptor (A2AR), inhibiting effector T cells [35]. Programmed cell death-1 (PD-1), expressed by activated effector Treg cells and effector T cells, may play a role in controlling Treg cell activation, although its exact effects on effector Treg cells are not yet fully understood.

#### TAMs

TAMs have been reported to be excessively present in the TME where they are responsible for various roles including immunosuppression, metastasis, angiogenesis, and more importantly tumor cell growth through induction of chemokine, cytokines, and growth factors [36, 37]. They are formed due to the extravasation of monocytes into the TME, which are then transformed into monocyte-derived TAMs. Different chemotactic factors including chemokines, cytokines, and growth factors facilitate the recruitment of monocytes to the TME. Notable ligands/receptors include CSF-1/CSF-1R, CCL2/CCR2, CX3CL1/CX3CR1, and vascular endothelial growth factor (VEGF)-A [38].

CSF-1 plays a role in the renewal of tissue-resident macrophages, recruitment, and M2 polarization of monocytes. The expression of high levels of CSF-1 has been found in gastric, breast, and esophageal cancers [39–41]. CCL2 is a potent chemokine that acts as a strong chemoattractant for CCR2+ monocytes. Its overexpression has been reported in liver, breast, prostate, and colorectal cancers. A couple of studies have found a correlation between the expression of CCL2 and the infiltration of TAMs in esophageal cancer and RCC [42, 43]. CX3CL1/CX3CR1+ is an axis that plays a crucial role in the recruitment of monocytes. It is involved in the recruitment of macrophages in breast cancer. VEGF-A mediates the recruitment of TAM to hypoxic regions of TME [38].

In cancer patients, a decrease in invasive CD8 T cells is linked to poor prognosis. TAMs play a pivotal role in suppressing these invasive T-cell activities. M2-like TAMs reduce the production of CXCL9 and CXCL10, which hampers the recruitment of CD8 T cells to the TME. M2-like TAMs directly hinder CD8 T-cell function by impeding their proliferation and activation through interactions with immune checkpoints [44, 45]. Tregs contribute to cancer's immune evasion by fostering an inhibitory TME. A positive feedback loop exists between Tregs and M2-like TAMs, which stimulate the activation of Treg cells originating from CD4, and CD25 T cells, and in reciprocation, these activated Treg cells promote the conversion of monocytes into an M2-like phenotype [46]. M2-like TAMs express immune checkpoint ligands such as programmed death ligand (PDL) 1, PDL2, B7-1, and B7-2, directly suppressing T-cell function. They also release cytokines like IL-10 and TGFβ, which reinforce a potent immunosuppressive microenvironment by inhibiting CD4 T and CD8 T cells and promoting the proliferation of Tregs [47]. TAMs nurture T-cell activity within the TME by depleting essential metabolites required for T-cell proliferation, producing anti-inflammatory cytokines like IL-10, TGF-β, and prostaglandin-E2 (PGE2) to hinder T-cell functions and engage inhibitory receptors like PD-1 and CTLA4, leading to T-cell dysfunction. In addition, TAMs upregulate the expression of PDL1, contributing to overall T-cell suppression, particularly in hypoxic tumor regions [48]. These multifaceted strategies employed by TAMs highlight their pivotal role in fostering an immunosuppressive environment within tumors.

#### TANs

Complex and multifaceted relationships between tumor cells and immune or non-immune stromal cells that favor cancer development and progression have been established by recent studies [49]. Although stromal cells within TME are genetically stable and are good cancer therapeutic agents, there has been increasing interest in studying the role of TANs in cancer development [49]. TANs are immunosuppressive cells that are documented to either promote or inhibit tumor progression in the TME [49, 50]. Their recruitment within tumors is orchestrated by a complex interplay of cytokines and chemokines, each contributing uniquely to the process while also influencing one another. Among cytokines, CXCL1 (GROα), IL-8, and tumor necrosis factor (TNF)-α play central roles in TAN recruitment.

CXCL1 and IL-8 act as potent chemoattractants for TANs. CXCL1, often overexpressed in tumors, functions by binding to its receptor on the surface of neutrophils, guiding them toward the tumor site [51]. Similarly, IL-8, produced by both tumor and stromal cells, exerts its chemoattractant effect on TANs by binding to its receptors [52]. These cytokines effectively create a directional gradient that steers TANs toward the tumor. TNF-α, a versatile cytokine within the TME, enhances TAN infiltration by promoting the expression of adhesion molecules on endothelial cells, facilitating the firm adhesion of circulating neutrophils to blood vessel walls, besides inducing the production of other chemotactic signals, indirectly amplifying the recruitment of TANs [53]. IL-1β contributes to TAN recruitment by upregulating endothelial cell adhesion molecules, enhancing the adhesion of TANs to the endothelium, and facilitating their subsequent extravasation into the tumor tissue [54].

In parallel with these cytokines, chemokines play a vital role in TAN recruitment. The chemokine receptor CXCR2, found on TANs, is pivotal for their migration into tumors. The binding of CXCR2 to its ligands, including CXCL1 and IL-8, triggers intracellular-signaling pathways that guide TANs toward the tumor site [55]. In addition, the proinflammatory cytokine IL-17A amplifies TAN recruitment by upregulating the expression of CXCR2 on neutrophils, enhancing their responsiveness to chemokines like CXCL1 and IL-8 [56]. This intricate network of cytokines and chemokines collectively shapes the recruitment of TANs within the tumor microenvironment, orchestrating their directional migration and ultimately influencing the tumor's immune milieu and progression.

Several studies have documented the immunosuppressive role of TANs during tumorigenesis. In the initial stages, innate and adaptive immune cells cooperate to combat small tumor cell clusters. However, in advanced tumor models, TANs have been observed to induce CD8 T-cell apoptosis through the TNFα pathway and NO, contributing to an immunosuppressive environment. TANs can also inhibit the activation of T cells, suppress their T-cell proliferation, and impair their antitumor functions through mechanisms like releasing arginase 1 (ARG1), ROs, and NO and by modulating the PDL1/PD-1-signaling pathway [57–59]. Furthermore, TANs are capable of suppressing NK cells as well as facilitating the recruitment of other immunosuppressive cells like Treg by initiating the release of CCL17 [14]. Unlike other stromal cells like fibroblasts and macrophages derived from CCL2 in the TME, TANs-derived CCL2 are found in high amounts which facilitates macrophage recruitment and infiltration of the tumor sites [49, 60]. Apart from CCL2, TAN-derived CCL17 (C–C chemokine) recruits CCR4þ and Treg cells for tumor progression. In vitro and in vivo studies by Zhou et al. [49] revealed the role of TANs in recruiting macrophages and Treg cells.

### Angiogenesis

After immune suppression and evasion, the tumor now requires blood rich in nutrients and oxygen for further proliferation and survival. Angiogenesis, the creation of new blood vessels from existing vasculature, is a physiologic process hijacked by the TME to ensure a continuous supply of the needed nutrients and oxygen for tumor survival [61]. This is achieved through combined cytokine and chemokine expression in the TME [61]. Angiogenesis is a complex phenomenon that is mediated through sequential steps. In general, the TME becomes hypoxic when the tumor size reaches approximately 2 mm. This induces hypoxia-inducible factor-1, consequently stimulating the release of pro-angiogenic cytokines and chemokines [61]. Hypoxia also upregulates the expression of proteases such as matrix metalloproteinases degrading the basement membrane and extracellular matrix, allowing pro-angiogenic factors to reach the vasculature [61]. Among pro-angiogenic cytokines and chemokines, VEGF and stromal cell-derived factor (CXCL12) have been extensively studied as potent mediators of angiogenesis. The endothelial effects of these two components are as follows; induction of proliferation and migration of endothelial cells, increased endothelial cell permeability, and inhibition of apoptosis allowing new endothelial cells to survive [61, 62]. All these ultimately result in the growth of new blood vessels into the tumor.

### Tumor growth and replication

**Fig. 3:**
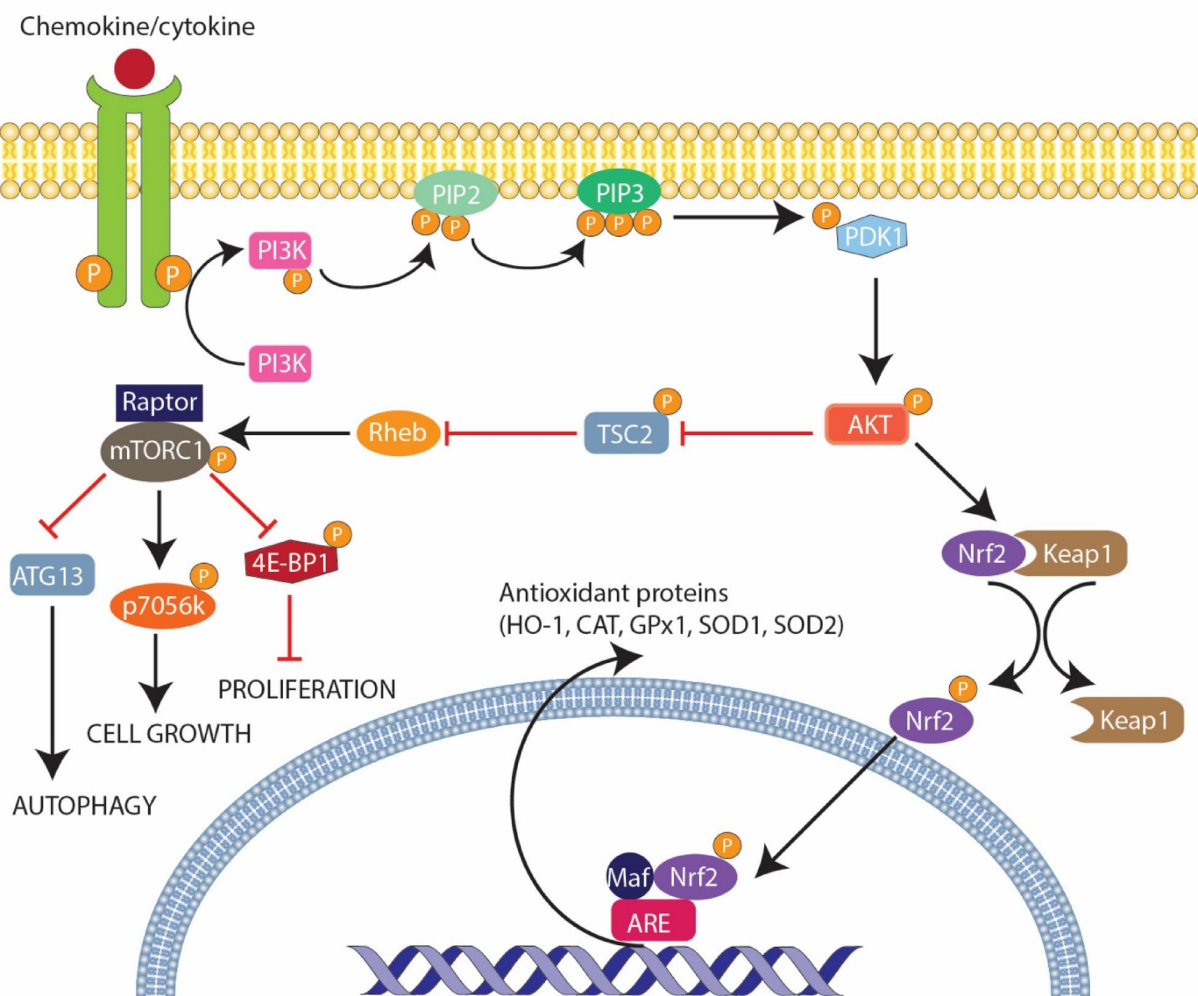
Mechanism of cytokine/chemokine activated PI3K/AKT-signaling pathway in tumor growth and replication. The PI3K/AKT pathway is a cellular signaling that leads to cell growth and survival when activated. Interestingly, some cytokines and chemokines have been found to utilize this pathway to promote tumor cell growth and replication

The creation of new blood vessels through angiogenesis allows additional nourishment to be delivered to the tumor for further growth, replication, and metastasis. Tumor growth and replication occur when physiological processes that regulate cell growth and division are disrupted. The primary regulatory processes are; the presence of proto-oncogenes (e.g. Cyclin-dependent kinase) and tumor suppressor genes (primarily retinoblastoma protein 1, p53, and p21). In addition, chemokines and cytokines such as; TNF-α, IL-1, IL-6, hepatocyte growth factor, Interferon-alpha (IFN-α), CCL-1, CCL-20, and CCL-25 promote tumor growth and replication by activating diverse-signaling pathways such as; phosphatidylinositol-3 kinase (PI3K/AKT), the mitogen-activated protein kinase /extracellular signal-regulated kinase MAPK/ERK 1/2, and the nuclear factor-kappa-β (NF-kB) pathways (Table 1), all of which are involved in cell proliferation, survival, and metastasis [63–66]. The PI3K/AKT pathway promotes cell survival and growth by inhibiting apoptosis and stimulating cell proliferation (Fig. 3) [67]. The ERK 1/2 pathway promotes cell growth and proliferation by activating transcription factors that control gene expression in cell growth and division [68]. The NF-kB-signaling pathway acts as a transcription factor that triggers the expression of genes that promote cell survival, proliferation, angiogenesis, and immune evasion [69, 70].

**Table 1:** Role of cytokines and chemokines in tumor growth and replication

	Mechanism of action
Cytokines	
TNF-α [71]	Activation of nuclear factor-kappa-β (NF-kB)-signaling pathways Stimulates production of cytokines (ex. IL-6) and growth factors Induces expression of adhesion molecules
IL-1 [72]	Activation of NF-kB-signaling pathways Activation of extracellular signal-regulated kinase 1/2 (ERK1/2) pathway Recruitment of immune cells to TME Stimulates production of angiogenic factors
IL-6 [72–74]	Activation of JAK-STAT3-signaling pathway Activation of PI3K/AKT-signaling pathway Recruitment of immune cells to TME Promotes epithelial–mesenchymal transition Stimulates production of angiogenic factors
HGF [75, 76]	Activates c-MET-related-signaling pathways (ex. Wnt/β-catenin signal transduction) Activation of PI3K/AKT-signaling pathway Activation of MAPK/ERK 1/2 pathway Activation of NF-kB-signaling pathways
IFN-α [77]	Activation of JAK-STAT3-signaling pathway Stimulates the production of IL-6 Upregulates expression of Bcl-2 and survivin Stimulates production of angiogenic factors
Chemokines	
CCL-1 [78]	Activation of PI3K/AKT-signaling pathway Activation of ERK1/2-signaling pathways
CCL-20 [78, 79]	Participates in CCL20–CCR6 axis enhancing migration and proliferation
CCL-25 [78, 80]	Upregulates expression of MMP2 and MMP9 through CCL25–CCR9 axis

### Metastasis

Tumor metastasis is a main prognostic indicator and is causative in 90% of cancer-related deaths [8]. Tumor metastasis is multifactorial and encompasses a complex interaction between tumor cells, extracellular matrix, adjacent tissues, and the vascular system. The process involves epithelial-to-mesenchymal transition (EMT), invasion of surrounding tissues to enter circulation, and spread to distant organs to initiate a new tumor [8, 66]. EMT is the most important property of tumor metastasis. It is mainly mediated by the proinflammatory cytokine TGF-β, further provoked by cytokines such as IL1B, TNF, IL-6, and NF-kB [8]. In addition, chemokines such as CXCL8, CCL5, and CCL18 have also been found to induce EMT [66]. Although poorly understood, it is a known fact that metastatic tumors exhibit organ-specificity, meaning they are more likely to target-specific organs. Accumulating evidence suggests that chemokines play a crucial role in tumorigenesis, by serving as a navigational tool for tumor cells that express corresponding receptors to migrate and locate to specific destinations [81]. Among the most widely studied chemokines, CXCL12–CXCR4 has been shown to facilitate organ metastasis. In a study by Müller et al. high expression of CXCR4 in malignant breast cancer cells has been found to control their chemotaxis towards its ligand CXCL12. Bone, lung, liver, and lymph node organs express high levels of CXCL12, which makes them preferred locations for malignant breast cancer cells to metastasize [82]. Other chemokine pairs, such as the CCL21–CCR7 and CXCL13–CXCR5 axis, have also been reported to facilitate breast and prostate cancer metastasis to the lymph node and bones, respectively [83, 84].

## Antitumor role of chemokines and cytokines

### Facilitation of antigen presentation

The activation, recruitment, and coordination of the immune system during tumorigenesis is multifaceted. It begins with the processing of tumor-associated antigens by antigen-presenting cells such as DCs (Fig. 1) [14]. During this process, DCs upregulate CCR7, facilitating their migration towards the tumor-draining lymph nodes where their ligands CCL9 and CCL21 are highly concentrated. This same chemokine axis facilitates the entry of naive CD8 and CD4 into the tumor-draining lymph node, while CCR5–CCL5 facilitates their interactions with DCs. Upon activation, T cells upregulate the expression of CXCR3 which drives their migration to interfollicular areas of the lymph node [14]. CXCR3 also facilitates secondary interactions of tumor-associated antigen (TAA)-specific CD4 T cells with DCs, which enables them to differentiate towards Th1 cells. This is achieved in the presence of cytokines such as IL-1B, IL-12, IL-18, and (IFN)-γ in the TME [85, 86]. For instance, IL-12 which is secreted by antigen-presenting cells due to direct immune intercellular contact [87], is reported to promote the differentiation of naïve CD4 Th0 cells into Th1 cells and the CD8 T cells into CTL. IL-12 has found considerable applications in cancer immunotherapy due to its established antitumor properties in preclinical models, hence bridging the gap between innate and adaptive immune systems [86].

### Recruitment and activation of specific immune cells in TME

#### NK cells

The recruitment, functionality, and trafficking of human and murine NK cells to the TME are coordinated by varieties of chemokines and chemokine receptor axes including CXCR1, CXCR3, CXCR4, CXCR6, CCR7, CCL (C–C motif chemokine ligand), CCL5, CXCL1 (C–X–C motif chemokine ligand 1), CCL5–CCR5, CCL27–CCR10, and CX3CL1–CX3CR1 [88, 89]. The infiltration of NK cells into the TME eliminates target cells as well as provides immunomodulatory cytokines which can enhance adaptive immune responses [90, 91]. Available clinical statistics show a correlation between the abundance of NK cells in the TME of human tumors and better outcomes in cancer patients including SCC of the lung, RCC, hepatocellular carcinoma, non-small cell lung cancer (NSCLC), pulmonary adenocarcinoma, gastric cancer, breast cancer, and melanoma [90, 92, 93].

The activation of NK cells with inherent antitumor immune response by certain cytokines like IL-2, IL-12, IL-15, IL-18, IFN-γ, and CCL-5 have been reported in preclinical model studies [94, 95]. Moreover, the secretion of various immune cells recruiting and antitumor response regulating cytokines like IFN-γ, granulocyte–macrophage colony-stimulating factor (GM-CSF), G-CSF, M-CSF, TNF, IL-5, IL-10, IL-13, FLT3LG, TGF-α, XCL, CCL3/4/5 by activated NK cells are well-documented in previous studies [88, 96]. Of all aforementioned cytokines, IFN-γ remains the most studied in shaping adaptive immune responses. This cytokine acts on a variety of immune cells such as DCs, T cells, and even NK cells [97]. The secretion of IFN-γ by NK cells is responsible for the direct elimination of cancer stem cells or undifferentiated tumors by NK cells [98]. The activation of NK cells does not only induce cytotoxicity towards target cells but also results in the secretion of chemokines and cytokines like IFN-γ making NK cells at the center of modulating the activity of other immune cells [99].

Furthermore, NK cells secrete abundant chemokines like CCL5 and XCL1/2 which are essential in recruiting an antitumor immune subset called conventional type 1 (cDC1) [100]. Interestingly, an AML model study reported that Innate lymphoid cells (ILC)-1 (NK-cell subset) are more effective in targeting leukemia stem cells than NK cells [101].

#### Dendritic and T cells

The infiltration of cDC1s is highly dependent on NK-cell-derived chemokines like XCL1 and CCL5 [102]. It is noteworthy to mention that tumor-infiltrating cDC1s are the major producers of CXCL9 and CXCL10 chemokine ligands that facilitate the recruitment of CD8+ T cells into the TME [103]. These chemokines offer antitumor activity by facilitating the accumulation of cDC1 and T-cell accumulation at the tumor core. Furthermore, the T-cell expansion and acquisition of effector functions can be promoted by the chemokines via interactions between cDC1 and CD8+ T cells [14]. Chemotherapeutic treatment of tumor-bearing mice was reported to lead to intratumoral expression of CXCL9, CXCL10, and CCL5 chemokines which facilitate recruitment and infiltration of CD4+ and CD8+ T cells into the tumor bed [104]. Similar chemokines were also observed in melanoma patients undergoing chemotherapy. The expression of these chemokines resulted in CD4+ and CD8+ T-cell infiltration, tumor control, and patient survival [104].

One of the cytokines that induce antitumor properties of T cells is IL-15, which is a four-alpha helix cytokine produced by antigen-presenting cells such as macrophages, monocytes, and other types of immune cells [94, 105]. The enhancement of T-cell proliferation and survival, proliferation, and differentiation of NK cells, and differentiation of CTL are the major immune stimulation effects of IL-15 that can result in anticancer properties (Table 2) [94]. Prolonged expansion and activation of NK cells and CD8 T cells by IL-15 cytokines have been posited in different studies [106]. IL-15 shares biological properties with IL-2; however, the application of IL-15 in antitumor therapy has been reported to be more advantageous compared to IL-2. Less expansion of Tregs and activation induces the death of effector T cells, which are some potential advantages of IL-15 compared to IL-2. In addition, the use of IL-15 in anticancer immunotherapy does not lead to serious toxicity emanating from major capillary leakages [107].

**Table 2:** Anti-tumor roles of cytokines during tumorigenesis

Cytokine	Secretory cells	Anticancer effects
IL-2 [77, 115]	CD4+ T cells, CD8+ T cells, NK cells, DCs, mast cells	↑ CD4+ T-cell differentiation, ↑ CD8+ T-cell cytotoxicity, ↑ T-cell proliferation, ↑ NK-cell proliferation and activation
IL-7 [116]	Thymic stromal and mesenchymal cells, lymphatic endothelial cells, intestinal epithelial cells	↑ T-cell, B-cell, and NK-cell proliferation
IL-12 [117]	DCs, phagocytes (monocytes/macrophages and neutrophils)	↑ CTL and NK-cell cytotoxicity; ↑ IFN-γ secretion by T cells, NK cells, ILCs; ↑ antigen presentation
IL-15 [118, 119]	DCs, monocytes, epithelial cells	↑ T-cell and NK-cell activation and proliferation
IL-21 [120, 121]	CD4+ T cells, NKT cells	↑ CD8+ T-cell, NK-cell, and NKT-cell cytotoxicity
IFN-α, IFN-β [122, 123]	Lymphocytes (NK cells, B cells, and T cells), macrophages, fibroblasts, endothelial cells, osteoblasts	↑ DC maturation and activation; ↑ MHC class I expression on tumor cells; ↑ NK-cell maturation and cytolytic effect
IFN-γ [85]	CD4+ T cells, CD8+ T cells, NK cells, B cells, NKT cells, professional antigen Presenting Cells (APCs)	↑ Cell surface MHC class I expression; ↑ T-cell, NK-cell, and NKT-cell migration into tumors

### Coordinating effector T-cell antitumor attack at tumor sites

The coordinated recruitment of various immune cells including effector T cells to target tissues or tumor sites accounts for successful immunity to different diseases including malignancies. Once coordinated recruitment is achieved, effector T cells leverage localized chemotactic cytokines from the tumor bed to infiltrate the tissue and interact with antigen-presenting cells to liberate effector cytokines [108]. The ability of the effector T cells to identify specific sites within cancerous tissues has been reported to be under the regulation of microanatomical display of chemotactic and adhesive signals. Immune surveillance and rapid response of effector functions by the coordinated immune cells within the TME are greatly facilitated by distinct spatial precision of the immune cells to avoid collateral damage to surrounding health tissues [109, 110]. The expression of CXCL9 chemokine in the tumor bed has been reported to enhance the proximity of CD8+ T cells to IL-12-producing cDC1s and consequently promote cytokine exposure and T-cell effector functionalities due to the ability of IL-12 to promote T-cell proliferation [111]. Similarly, the expression of CXCL9 and IL-12 by cDC1s has been reported to enhance immunotherapy response against PD-1. To enhance the expression of these chemokines and cytokines by tumor cDC1s, increased production of IFNγ by effector T cells has been suggested [112, 113]. However, the role of CXCL9 in promoting CD8+ T-cell localization and exposure to IL-12 and increasing IFNγ production without a corresponding IFNγ by cDC1s remains to be evaluated [111, 114].

## Chemokines and cytokines in cancer therapy

Over the past several decades, radiation therapy, surgery, and chemotherapy have been the mainstay of anticancer therapy. However, limitations such as collateral damage to normal tissues, and the inability to treat minimal and metastatic residual disease have led to the development of novel avenues for cancer treatment, such as immunotherapy [10]. Several strategies, such as immune checkpoint inhibitors, monoclonal antibodies against tumor antigens, and adoptive cell therapies, are currently being developed in the field of immunotherapy. However, the chemokine and cytokine systems have gained more importance over the past few years as promising drug targets in cancer immunotherapy due to their influence on various processes involved in various aspects of tumor biology.

Many cytokines, including GM-CSF, IL-7, IL-12, IL-15, IL-18, and IL-21, have entered clinical studies for people with advanced cancer [124]. However, only two cytokines have received FDA (Federal Drug Administration) approval as single anti-cancer agents so far: IFN-alpha for Stage III melanoma adjuvant therapy and IL-2 for metastatic melanoma and RCC [125–127]. Moreover, combining these cytokines with different checkpoint inhibitors has been suggested to improve the effectiveness of checkpoint inhibition, re-establishing the role of cytokines in cancer immunotherapy. Early studies using IL-2 and IFN- to block Cytotoxic T-lymphocyte-associated protein 4 (CLTA4) and PD-1/PDL1 have been completed with encouraging results [128, 129].

The remarkable efficacy of chimeric antigen receptor (CAR) T-cell therapy in treating hematological malignancies has been overshadowed by challenges such as cytokine release syndrome (CRS) and immune effector cell-associated neurotoxicity syndrome (ICANS) [130]. Understanding the underlying mechanisms of these toxicities and devising effective prevention and treatment strategies have become paramount. Recent advancements have focused on harnessing cytokines to bolster CAR T-cell functionality and enhance cytotoxicity against solid tumors, which often possess hostile microenvironments [131]. Due to promising efficacy outcomes, anti-CD19 CAR T cells have gained FDA approval, showcasing their potential in treating hematologic malignancies. Alongside this, the utilization of tumor-infiltrating T lymphocyte cultures and adoptive T-cell therapies is also maturing, further advancing the field of immunotherapy [132, 133]. Notably, the success of in vitro activation and in vivo survivability of transplanted T cells relies significantly on cytokines. Cytokine genes can be strategically integrated into the lentiviral vector that encodes the CARs, leading to improved cellular immunotherapies [134]. To address these challenges, researchers have developed fourth-generation CAR constructs that release cytokines, thereby augmenting T-cell cytotoxicity and improving therapeutic outcomes. Integrating cytokines into the manufacturing process of CAR T cells has further facilitated their expansion and differentiation, ultimately enhancing treatment efficacy [131]. In B-cell acute lymphoblastic leukemia (B-ALL) treatment, CAR-T therapy has shown exceptional clinical outcomes, yet challenges persist, including CRS, neurotoxicity, and B-cell aplasia-associated hypogammaglobulinemia [135]. However, with continuous research, improvements in media design, and other phenotype-determining factors, CAR-T therapy shows great promise for B-ALL and other hematological malignancies [135].

The role of the gut microbiome in modulating cytokine release syndrome and therapeutic responses in CAR-T therapy has also been investigated, highlighting its impact on treatment outcomes. Certain genera in the gut microbiome were found to correlate with clinical responses to CAR-T therapy, indicating a potential avenue for optimizing therapeutic outcomes [136]. With the FDA approval of various CAR-T-cell therapies, including Kymriah, Yescarta, and Tecartus, significant progress has been achieved in the field [137]. In addition, the establishment of clinical research and consensus guidelines for managing CRS and neurotoxicity has further enhanced the safety and efficacy of CAR-T-cell therapy [138]. As we move forward, integrating cytokines and exploring the gut microbiome's influence promise to unlock even greater potential in CAR T therapy, solidifying its position as a promising and transformative approach to cancer treatment.

With regards to the use of chemokines in cancer therapy, Table 3 summarizes various targets, inhibitors, tumor models, and mechanisms of action for chemokine receptors, which are increasingly being investigated for their potential in cancer treatment.

**Table 3:** Chemokine and chemokine receptor inhibitors in cancer immunotherapy

Target	Inhibitor	Tumor model	Mechanism of action
CCR1 [139, 140]	CCX721 BL5923	Multiple Myeloma Hepatic spread of Colon Cancer	Blocks excess osteoclast activity Suppresses metastatic colonization of myeloid cells
CCR2 [141]	CCX872 + Anti-PD1	Pancreatic cancer	Enhances the therapeutic effect of Programmed cell death protein ligand 1 (PDL1) immunotherapy
CCL2 [142]	CNTO 888 + Radiotherapy	Breast Cancer	Inhibits CCL2-induced calcium mobilization, inhibits angiogenesis and improves the impact of radiotherapy
CCR4 [143, 144]	Mogamulizumab Anti-CCR4 CAR-T Cells	Relapsed adult T-cell leukemia T-cell malignancies	Blocks CCR4-mediated signal transduction pathways and chemokine-mediated angiogenesis Increases the number of natural killer cells and changes the phenotype of myeloid cells into anti-tumorigenic
CCR5 [145–147]	Maraviroc TAK-779	Colorectal Cancer Melanoma and Pancreatic cancer	Decreases rate at which fibroblasts associated with cancer accumulates + suppress cellular growth in leukemia model Inhibits Ligand Binding to CCR5
CCR7 [148, 149]	let-7a (siRNA)	Prostate and Colorectal cancer	Directly binds to the 3'UTR of CCR7 and blocks its protein expression
CXCR2 [150]	Riparixin + PTX	Breast Cancer	Inhibits CXCL8 receptors CXCR1 and CXCR2, reducing intracellular signaling, breast cancer stem cells, and metastases formation

## Current challenges associated with the use of cytokines and chemokines in cancer therapy

The advancements in cytokine and chemokine-targeted therapies have significantly underscored the potential of immunotherapies in the field of oncology. Cytokine-based immunotherapies have faced several issues, which are broadly categorized into high toxicity and low efficacy. The high toxicity of cytokine therapies is a significant challenge that limits their clinical use. Many cytokine therapies can cause severe side effects such as flu-like symptoms, fatigue, organ dysfunction, and even life-threatening conditions like capillary leak syndrome. These dose-limiting toxicities can lead to the discontinuation of therapy, which ultimately limits their clinical efficacy [87].

High-dose interleukin-2 (IL-2) therapy has achieved long-term remission in a small percentage of patients with advanced melanoma and renal cell carcinoma. However, the treatment is associated with severe toxicities that limit its broader use [151]. Cytokine therapies can cause significant side effects that can be dose-limiting. For example, IL-2 can cause capillary leak syndrome, leading to fluid retention, hypotension, and organ dysfunction [151]. IFN-α can cause flu-like symptoms, depression, and fatigue [152].

Moreover, the pleiotropic nature of cytokine signaling is another significant hurdle to overcome for optimal use in immunotherapy. Cytokines have dual immunosuppressive and immunostimulatory functions that can be redundant, limiting their efficacy as a monotherapy. In addition, cytokines can interact with multiple cell types in the TME, including immune cells, cancer cells, and stromal cells, which can influence cytokine function and the overall outcome of therapy [153]. Cytokines have pleiotropic effects, meaning they can act on multiple cell types and have both pro- and anti-inflammatory effects. This lack of specificity can limit their efficacy and increase the risk of side effects. For example, IL-2 can activate regulatory T cells, suppressing the immune response and limiting its antitumor activity [151]. The TME is complex and dynamic, with multiple cell types and signaling pathways interacting to promote tumor growth and immune evasion. This complexity can limit the efficacy of cytokine therapies, as the cytokines may not be able to penetrate the TME or may be counteracted by other signaling pathways [154]. The efficacy of cytokine therapies may also vary depending on the specific tumor type, stage, and genetic makeup of the patient, further highlighting the challenges of using cytokines as immunotherapy for cancer.

Similarly, chemokine receptors and their corresponding ligands hold great promise as therapeutic targets, owing to their influential regulatory functions in both cancerous and infiltrating immune cells. Nonetheless, developing treatments that modulate the TME is an intricate task, and chemokine-directed therapies pose a particularly challenging obstacle due to their multifaceted and occasionally contradictory roles in tumorigenesis.

The administration of small-molecule inhibitors or antibodies that target chemokine receptors or ligands may lead to unforeseen adverse effects, as all cells expressing the targeted chemokine or receptor could potentially be impacted. However, the use of Mogamulizumab, which targets CCR4, is associated with fewer concerns in this regard, as only a subset of T lymphocytes expressing CCR4, such as Th2, Tregs, and Th17 cells, are affected. Of note, Treg and Th17 cells possess immunosuppressive characteristics, therefore their depletion could have beneficial implications in the context of cancer treatment [155].

The long-term depletion of Treg cells resulting from Mogamulizumab treatment is associated with an increased risk of severe skin lesions and worsening of graft-versus-host disease in patients who have undergone bone marrow transplants. This highlights a significant challenge in the development of chemokine-targeted therapies, particularly for chemokine receptors that have less variability in their expression compared to CCR4. Targeting receptors that are expressed by a large proportion of leukocytes, such as CXCR4 or CCR7, carries a greater risk of unpredictably affecting the host's immune response and could lead to serious immune-mediated side effects [156].

The interdependent relationship between chemokines and the host immune system represents a significant limitation of chemokine-targeted therapies. The inhibition of a specific chemokine-receptor axis could result in either advantageous or deleterious effects on disease progression, acting as either a tumor suppressor or promoter, depending on the type, stage, and immunological landscape of the tumor [157].

Chemokine-targeted therapies impact tumor-infiltrating cells, as shown by CCR2+ cell data in mouse models. Anti-CCR2 therapy may promote metastatic spread if macrophages infiltrate, but it could be detrimental if CD8+ T-cells enhance immunosurveillance. Ma et al. found that anthracycline-based chemotherapy activates DCs and T-lymphocytes, recruiting specialized antigen-presenting cells within the tumor, independent of draining lymph nodes. Increased Ccl2 expression was observed in tumors treated with anthracyclines, crucial for recruiting dendritic cell-like APCs. Lack of Ccl2 or Ccr2 reduced therapeutic responses, highlighting CCL2/CCR2 axis importance for immunogenic chemotherapy's efficacy [158].

Incorporating chemokine-targeted therapies alongside conventional medical care and immunotherapies may heighten the likelihood of adverse immunological responses, a widely adopted strategy in clinical trials. While extensive literature highlights the synergistic benefits of these treatments when administered alongside other drugs, the combination of multiple cancer therapies can potentially elevate toxicity levels, even though it may also enhance efficacy.

Notably, the challenges in creating and transferring novel chemokine-related target therapies are particularly exacerbated by the dearth of suitable animal models that accurately reflect the characteristics and behavior of human tumors [159]. Preclinical models are used in the clinical translation of cancer immunotherapy to prioritize drug targets and examine drug mechanisms of action, delivery strategies, treatment regimens, doses, and safety [160]. Cancer-induced models frequently fall short in their attempts to replicate the diversity and complexity of the networks that connect human immune cells and malignancies, and they have repeatedly failed to predict clinical success rates [161–163].

The aforementioned challenges underscore the necessity of determining the optimal therapeutic range for each chemokine target and various cancers, to achieve an antitumoral effect while minimizing adverse effects on the host's immune system. Moreover, efforts are being made to enhance the preclinical models to enhance tumoral immunogenicity through the utilization of humanized mouse models, genetically engineered mouse models, organoids, and mammospheres derived from human tumor stem cell precursors, as well as ex vivo technology and alternative animal models that are more closely related to human biology [164]. This will contribute to the advancement of immuno-oncology research and improve the success rate of preclinical testing of immune-based cancer therapies.

## Recommendations and future outlook

Chemokines and cytokines can potentially affect chemotaxis and cell migration, thus to better assess the bidirectional regulation, co-culture studies are needed that are composed of cancer cells and other immune cells. Different types of cells, like fibroblasts and endothelial cells, must also be included to observe their role. Various clinical trials must be conducted to further understand the role and effect of chemokines and cytokines. After establishing a prognostic role, further studies must be conducted to determine the underlying mechanism that will help to develop cheap diagnostic and management techniques. Random migration and chemotaxis should be studied using different modalities to understand the migratory pathways better. Using three-dimensional systems can mimic the physiological characteristics of the environment around a tumor instead of traditional two-dimensional means. Radiomics and AI offer promise in improving renal lesion characterization and treatment prediction. Challenges include variable feature selection, image acquisition standardization, and limited validation across patient cohorts, hindering clinical translation [165]. Efforts must focus on refining methods and validation frameworks to ensure reliability in practice. Integrating these advancements with immunotherapy could revolutionize cancer treatment by addressing existing limitations and enhancing patient outcomes.

Evidence suggests thyroid hormones (THs) interact with androgen receptors (ARs), regulating gonadal differentiation and reproductive function. THs increase AR expression, affecting androgen biosynthesis enzymes, and interact with TH-related genes. This crosstalk may influence prostate cancer development [166]. Periprostatic adipose tissue (PPAT) from prostate cancer patients enhances androgen-independent prostate cancer cell migration via connective tissue growth factor (CTGF) upregulation. Inhibition of the TGFβ receptor counters migration, suggesting therapeutic potential in targeting adipocyte-released factors and the TGFβ/CTGF axis against advanced prostate cancer [167]. Understanding the interplay between THs, ARs, and PPAT factors could refine clinical approaches to Prostate cancer management.

A vexing problem in cancer immunotherapy is how to turn cold tumors into hot ones. Cold tumors are tumors that lack effector T cells or contain an accumulation of Tregs that suppress the activity of effector T cells in the TME, while hot tumors are those that show signs of inflammation as a result of infiltration and antitumor attacks mediated by effector T cells in the TME. It is believed that tumor tumors do not respond to anticancer agents such as immune checkpoint inhibitors [168]. Cytokine/chemokine injection is a form of immunotherapy aimed at converting "cold" tumors into "hot" tumors [169, 170]. In this approach, specific cytokines and chemokines are directly injected into the tumor site to modify the TME and attract immune cells, thereby stimulating an anti-tumor immune response. Future studies could focus on identifying relevant cytokines and chemokines to enhance this approach. A study by Karin suggests the potential of CXCL10 and CXCL9-based therapies to turn cold tumors into hot and enhance antitumor activity; however, future clinical trials are required to determine if any of these could be used for cancer immunotherapy either as monotherapy or combination with immune checkpoint inhibitors [168].

## Study limitations

Despite our diligent efforts to conduct a comprehensive review, several limitations should be considered when interpreting the findings. Firstly, while we employed a thorough search strategy across multiple reputable databases, the possibility of missing relevant studies remains, which may introduce an inadvertent selection bias. In addition, our review was confined to English-language literature, potentially excluding valuable insights from non-English publications and introducing a language bias. The quality and availability of data on this intricate topic were variable, limiting the depth and breadth of our analysis. Data gaps, inconsistencies in data collection, and potential reporting bias in the reviewed studies further underscore the need for caution when interpreting the synthesized findings. Biases associated with the review process, such as selection and interpretive biases, cannot be entirely ruled out. Lastly, the geographical origin of the included studies may introduce a potential geographical bias, potentially limiting the generalizability of our findings across diverse populations. These limitations emphasize the complexities inherent in this field and the importance of future research endeavors to address these challenges.

## Conclusion

Ultimately, this review aimed to explore the distinctive abilities that cytokines and chemokines hold towards regulating immune responses. The dual roles of these proteins assist in driving the death of abnormal cells and regulating healthy cells by providing anti-cancer signals to the cancer-immune cell synapse. However, on the other hand, these same proteins may aggregate tumor development even further, primarily through stimulating inflammation and angiogenesis. The current advent of immunotherapeutic approaches for tumors focuses on up or downregulating mechanisms of the immune system. Therefore, devising cytokine and chemokine-derived targeted therapies poses useful interventions to target chemokine networks and high-affinity receptors, respectively. However, the development of these therapies is not without its challenges. Thus, to build fruitful novel therapies, tumoral immunogenicity and in vivo studies must be improved and customized to reflect the TME best. In addition, further research must be undertaken to assess the impact of these therapies' effects on overall cancer biology based on the cancer type being investigated.

## Data Availability

No datasets were generated or analysed during the current study.

## Abbreviations

B-ALL: B-cell acute lymphoblastic leukemia
CAR: Chimeric antigen receptor
cDC1s: Conventional Type 1 dendritic cells
CRS: Cytokine release syndrome
CTLA: Cytotoxic T lymphocytes antigen
DCs: Dendritic Cells
EMT: Epithelial-to-mesenchymal transition
FDA: Federal Drug Administration
GM-CSF: Granulocyte–macrophage colony-stimulating factor
IFN: Interferon
IL: Interleukin
MAPK/ERK: Mitogen-activated protein kinase/extracellular signal regulated kinase
M-MDSCs: Monocytic MDSCs
MDSCs: Myeloid-derived suppressor cells
NK: Natural killer
NF-kB: Nuclear factor-kappa-beta
PI3/AKT: Phosphatidylinositol-3 kinase
PMN–MDSCs: Polymorphonuclear–MDSCs
PD-1: Programmed death-1
PDL: Programmed death ligand
ROS: Reactive oxygen species
RCC: Renal cell carcinoma
STAT 3: Signal transducer activation of transcription 3
TAMs: Tumor-associated macrophages
TANs: Tumor-associated neutrophils
TME: Tumor microenvironment
TGF: Transforming growth factor
Treg: Regulatory T
Th: T Helper
TNF: Tumor necrosis factor
VEGF: Vascular endothelial growth factor
